# Characterization of *PHGDH* expression in bladder cancer: potential targeting therapy with gemcitabine/cisplatin and the contribution of promoter DNA hypomethylation

**DOI:** 10.1002/1878-0261.12697

**Published:** 2020-06-20

**Authors:** Hirofumi Yoshino, Hideki Enokida, Yoichi Osako, Nijiro Nohata, Masaya Yonemori, Satoshi Sugita, Kazuki Kuroshima, Masafumi Tsuruda, Shuichi Tatarano, Masayuki Nakagawa

**Affiliations:** ^1^ Department of Urology Graduate School of Medical and Dental Sciences Kagoshima University Kagoshima Japan; ^2^ MSD K.K Tokyo Japan

**Keywords:** apoptosis, bladder cancer, GC therapy, methylation, PHGDH

## Abstract

d‐3‐Phosphoglycerate dehydrogenase (PHGDH) conducts an important step in the synthesis of serine. Importantly, the *PHGDH* gene is often amplified in certain cancers. Our previous studies revealed that *PHGDH* gene amplification was associated with poor overall survival in clear cell renal cell carcinoma (ccRCC) and that metabolic reprogramming of serine synthesis through PHGDH recruitment allowed ccRCC cells to survive in unfavorable environments. There have been no investigations of the role of *PHGDH* expression in bladder cancer (BC). In this investigation, we examined the clinical importance of PHDGH in BC. Furthermore, we asked whether PHGDH expression could be exploited for BC therapy. Finally, we investigated the regulatory mechanisms that modulated the expression of PHGDH. Using data from The Cancer Genome Atlas, we found that patients with high‐grade BC had significantly higher *PHGDH* expression levels than did those with low‐grade BC. In addition, patients with high *PHGDH* expression did not survive as long as those with low expression. *PHGDH* downregulation by si‐RNAs or an inhibitor in BC cell lines significantly inhibited proliferative ability and induced apoptosis. Furthermore, combined treatment using a PHGDH inhibitor and gemcitabine/cisplatin achieved synergistic tumor suppression compared to use of a single agent both *in vitro* as well as *in vivo*. Mechanistic analyses of *PHGDH* regulation showed that *PHGDH* expression might be associated with DNA copy number and hypomethylation in BC. These findings suggest novel therapeutic strategies could be used in BC. Finally, our data enhance our understanding of the role of PHGDH in BC.

## Graphical abstract

This research investigated PHGDH, a key enzyme in the serine biosynthesis, in bladder cancer. PHGDH downregulation significantly inhibited cell proliferation, and the combined treatment between PHGDH inhibitor and gemcitabine/cisplatin achieved synergistic tumor suppressive effect compared to each single agent group both *in vitro* and *in vivo*. We also indicated that PHGDH was regulated by DNA copy number and hypomethylation.

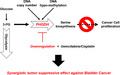

**Abbreviations**
BC: bladder cancer
ccRCC: clear cell renal cell carcinoma
GC: gemcitabine and cisplatin
PHGDH: d‐3‐phosphoglycerate dehydrogenase
SAM: *S*‐adenosylmethionine
TCGA: The Cancer Genome Atlas
TdT: terminal deoxynucleotidyl transferase

## 1 Introduction

Bladder cancer (BC) is a frequent cause of death around the world (Antoni *et al*., 2017). Siegel *et al*. (2018) estimated that there were more than 81 000 BC patients in 2018, of whom more than 17 000 died in 2018 in the United States alone. BC consists of two types of disease: those that are nonmuscle‐invasive (NMIBC) and those that are muscle‐invasive (MIBC). The former constitute 70–80% of BC patients (Miller *et al*., 2016). The remaining patients diagnosed with localized MIBC are treated by radical cystectomy or radiotherapy, but they have poor outcomes. Half of the patients undergoing surgery show metastasis in under 2 years (Sternberg *et al*., 2013). Experience has shown that treatment of advanced BC with gemcitabine and cisplatin (GC) has limited efficacy, as the response rates are only 50% (Kaufman *et al*., 2009; Sternberg *et al*., 2013). Importantly, high rates of severe toxicities and inherent or acquired drug resistance are often observed (Bergman *et al*., 2002; Wang and Lippard, 2005). Based on data from the United States (Abdollah *et al*., 2013), the 5‐year survival rate following such treatment has only improved slightly in the last three decades. Treatment with antiprogrammed death‐1 (PD‐1) antibodies constitutes a different approach to advanced BC. However, the response rate was only 21.1%, and overall survival was enhanced by only a few months (Bellmunt *et al*., 2017). In addition, there are no reliable markers to indicate metastasis or recurrence. Therefore, new treatment options to increase chemosensitivity and novel prognostic markers are needed.

In the glycolytic scheme, d‐3‐phosphoglycerate dehydrogenase (PHGDH) is an oxido‐reductase that contributes to the biosynthesis of serine. To maintain rapid, sustained, and uncontrolled cell proliferation, cancer cells require serine, as it leads to one‐carbon units that contribute to *de novo* synthesis of purines and pyridines (Amelio *et al*., 2014). Importantly, the serine synthetic pathway converts about half of the cell's glutamate to α‐ketoglutarate when PHGDH is expressed at high levels. Therefore, PHGDH inhibition leads to a significant reduction of the tricarboxylic acid cycle (Amelio *et al*., 2014). Recently, it has been reported that *PHGDH* is amplified or overexpressed in various types of cancers (Locasale *et al*., 2011). Thus, it might offer a therapeutic target in both breast cancer and melanoma. We previously reported that metabolic reprogramming of serine synthesis thorough PHGDH recruitment was observed when hypoxia‐inducible factor 2a was knocked out in sunitinib‐resistant renal cell carcinoma. In addition, when the *PHGDH* gene was amplified, patients showed poorer (disease‐free survival) relative to patients lacking amplification (Yoshino *et al*., 2017a). Therefore, PHGDH might be a potential therapeutic target and biomarker for several cancers, a possibility that has not been investigated in BC.

In this study, we evaluated the clinical relevance of *PHGDH* in BC, and the efficacy of PHGDH inhibition in BC. We also evaluated the efficacy of combined PHGDH inhibition and GC treatment *in vitro* as well as *in vivo*. In addition, regulatory mechanisms associated with PHGDH in BC were analyzed, as this issue has not been examined in BC previously.

## 2 Materials and methods

### 2.1 Cell culture

The human BC cell line BOY (Yoshino *et al*., 2011) was established by our group. It originated from a 66‐year‐old Asian patient. He had been diagnosed with stage III BC with lung metastasis. Lines T24, KK47, J82, UMUC, MCF7, and MDAMB231 were purchased from the American Type Culture Collection (ATCC, Manassas, VA, USA). The cells were cultured for less than 30 continuous passages. The lines were found negative for mycoplasma (e‐Myco Mycoplasma PCR Detection Kit; iNtRON, Kyungki‐Do, Korea). These cell lines were maintained in minimum essential medium containing 10% FBS in a humidified atmosphere of 5% CO_2_ and 95% air at 37 °C. To check whether or not PHGDH expression was decreased by methylation, BC cells were treated with *S*‐adenosylmethionine (SAM) (B9003, New England Biolabs, Inc., Beverly, MA, USA) for 72 h.

### 2.2 RNA extraction and quantitative real‐time reverse transcription polymerase chain reaction

The Isogen (Nippon Gene, Tokyo, Japan) kit was used for extraction of total RNA following the manufacturer's protocol. A SYBR‐green quantitative PCR‐based array approach was used here as described (Yoshino *et al*., 2017b). The following primers were used: *PHGDH*, forward primer, 5′‐CTGCGGAAAGTGCTCATCAGT‐3′ and reverse primer, 5′‐TGGCAGAGCGAACAATAAGGC‐3′; *PSAT1*, forward primer, 5′‐TGCCGCACTCAGTGTTGTTAG‐3′ and reverse primer, 5′‐GCAATTCCCGCACAAGATTCT‐3′; *PSPH*, forward primer, 5′‐GAGGACGCGGTGTCAGAAAT‐3′ and reverse primer, 5′‐GGTTGCTCTGCTATGAGTCTCT‐3′; *SHMT1*, forward primer, 5′‐CTGGCACAACCCCTCAAAGA‐3′ and reverse primer, 5′‐AGGCAATCAGCTCCAATCCAA‐3′; and *GUSB*, forward primer, 5′‐CGTCCCACCTAGAATCTGCT‐3′, and reverse primer, 5′‐TTGCTCACAAAGGTCACAGG‐3′.

### 2.3 Immunoassays

The method of immunoblotting was previously described (Yoshino *et al*., 2017b). We used anti‐PHGDH antibodies (1 : 1000) (HPA021241; Sigma, St. Louis, MO, USA), anti‐Ki‐67 antibodies (1 : 1000, ab92742; Abcam, Cambridge, UK), anticleaved‐PARP antibodies (1 : 500, #5625; Cell Signaling Technology, Danvers, MA, USA), and anti‐β‐actin antibodies (1 : 2000, bs‐0061R; Bios, Woburn, MA, USA).

### 2.4 Assessment of cell growth, apoptosis, and colony formation

Loss‐of‐function experiments made use of *PHGDH* si‐RNA (catalogue nos. SASI_Hs01_00041882 and SASI_Hs01_00041884; Sigma) and negative‐control si‐RNA (D‐001810‐10; Thermo Fisher Scientific, Waltham, MA, USA). PHGDH inhibitors included CBR‐5884 (Focus Biomolecules, Plymouth Meeting, PA, USA) and NCT‐503 (AOBIOUS, Gloucester, MA, USA) as previously described (Pacold *et al*., 2016). DMSO was used to dilute both inhibitors following the manufacturer's recommendations. Cell proliferation was determined with XTT assays (Roche Applied Science, Tokyo, Japan) according to the manufacturer's instructions. Cell apoptosis was measured by flow cytometric determination using the CytoFLEX analyzer (Beckman Coulter, Brea, CA, USA) and a FITC Annexin V Apoptosis Detection Kit (BD Biosciences, Bedford, MA, USA) as per the manufacturer's recommendations. The positive control utilized 5 μg·mL^−1^ cycloheximide (Sigma). Colony formation assays were previously described (Yoshino *et al*., 2017b).

### 2.5 Lentivirus‐mediated gene expression

Lentivirus was used to achieve overexpression of PHGDH in cells, using a gift (pLJM5‐WT PHGDH) provided by D. Sabatini (Addgene plasmid #83901) (Pacold *et al*., 2016). We produced lentivirus as previously described (Yoshino *et al*., 2017b).

### 2.6 *In vivo* tumor xenograft model

A 100 µL suspension of 4 × 10^6^ BOY cells was combined with 100 µL Matrigel Matrix (Corning, Bedford, MA, USA). The mixture was used for subcutaneous injection into the sides of female nude mice (BALB/c nu/nu, 6‐ to 8‐week‐old). Mice were separated into four groups: vehicle, GC [gemcitabine 150 mg·kg^−1^, intraperitoneal (i.p.) injection, days 7 and 14, cisplatin 6 mg·kg^−1^, i.p., days 6 and 13], NCT‐503 (40 mg·kg^−1^, 5 times a week 1 day after tumor injection), or the combination of GC and NCT‐503. The weight of each mouse was used to normalize the dose and the injection volume was < 150 μL. The tumor fraction was used to conduct terminal deoxynucleotidyltransferase‐mediated dUTP‐biotin nick end labeling (TUNEL). All the animal experiments were approved by the animal care review board of Kagoshima University (approval no. MD17047).

### 2.7 TUNEL

Following xenografting, apoptotic cells in the tumor fraction were detected by the TUNEL method with a MEBSTAIN Apoptosis Kit Direct (code 8445 MBL, Woburn, MA, USA) per the directions of the manufacturer. Briefly, tumor fractions from mice were fixed with 4% paraformaldehyde, embedded in paraffin, and sectioned at 5‐μm intervals. Deparaffinized sections were incubated with 50 µL of terminal deoxynucleotidyl transferase (TdT) buffer II for 10 min at room temperature, followed by addition of 45 μL of TdT buffer II, 2.5 μL of FITC‐dUTP, and 2.5 μL of TdT. Incubation continued for 1 h at 37 °C in TB buffer for 15 min followed by four washes in PBS. Apoptotic cells were examined with a fluorescent microscope (Keyence, Osaka, Japan).

### 2.8 Analysis of a bladder cancer cohort with The Cancer Genome Atlas and the Gene Expression Omnibus

The Cancer Genome Atlas (TCGA) cohort database provided mRNA expression Z‐scores that had been generated by RNA sequencing. It included 412 patients with bladder urothelial carcinoma (BLCA or BC) for analysis of clinical relevance (2014; Cerami *et al*., 2012). Gene expression quantification used RSEM (Li and Dewey, 2011). Epigenomic, transcriptomic, and clinical information of TCGA BLCA and mRNA expression analyses from 967 cancer cell lines were acquired from TCGA (https://tcga‐data.nci.nih.gov/tcga/) and cBioPortal (http://www.cbioportal.org/public‐portal/) on June 1, 2018. For validation of the prognostic value of *PHGDH* mRNA expression, we employed PrognoScan (http://www.prognoscan.org/) (Mizuno *et al*., 2009), a database for meta‐analysis of the prognostic value of genes, using GSE13507 as an independent cohort of BC. Demographic characteristics for TCGA BLCA and GSE13507 cohorts categorized based on PHGDH expression level are shown in Tables S1 and S2, respectively. We analyzed the correlation between gene expression and DNA methylation in the first intron of *PHGDH* on chromosome 1, using data from the cg14476101 probe on Illumina (Illumina, Inc. San Diego, CA) Infinium HumanMethylation450K array in TCGA BLCA database. To study the correlation between PHGDH, PSAT1, PSPH, or SHMT1 mRNA expression and PHGDH copy number, TCGA BLCA database was also used. Web‐based enrichment pathway analysis by Enrichr (http://amp.pharm.mssm.edu/Enrichr/) (Kuleshov *et al*., 2016) was performed with the HumanCyc metabolic pathway term database (Romero *et al*., 2005).

### 2.9 Statistical analysis

The Mann–Whitney *U*‐test was performed to assess the statistical relationship between two groups. The Bonferroni‐adjusted Mann–Whitney *U*‐test was applied to examine the relationships among three variables and numerical values. To evaluate the correlation between two variables, we applied Spearman's rank test. BC patients' overall and disease‐free survivals (TCGA cohort) were assessed with the Kaplan–Meier method and log‐rank test. To establish independent factors for overall and disease‐free survival, we utilized a multivariate Cox proportional hazards model. The calculations were performed by expert stat view software, version 5.0 (Cary, NC, USA). For enrichment analysis, Enrichr computes the *P*‐values by Fisher's exact test, followed by the Benjamini–Hochberg procedure to correct for multiple hypotheses, and calculates the adjusted *P*‐values. More detailed information is described on Enrichr's website.

### 2.10 Ethics and standards for conducting of human and animal research

The experiments were undertaken with the understanding and written consent of each subject. The study methodologies conformed to the standards set by the Declaration of Helsinki. The study methodologies were approved by the ethics committee of Kagoshima University.

## 3 Results

### 3.1 *PHGDH* expression in BC: the clinical significance

**Fig. 1:**
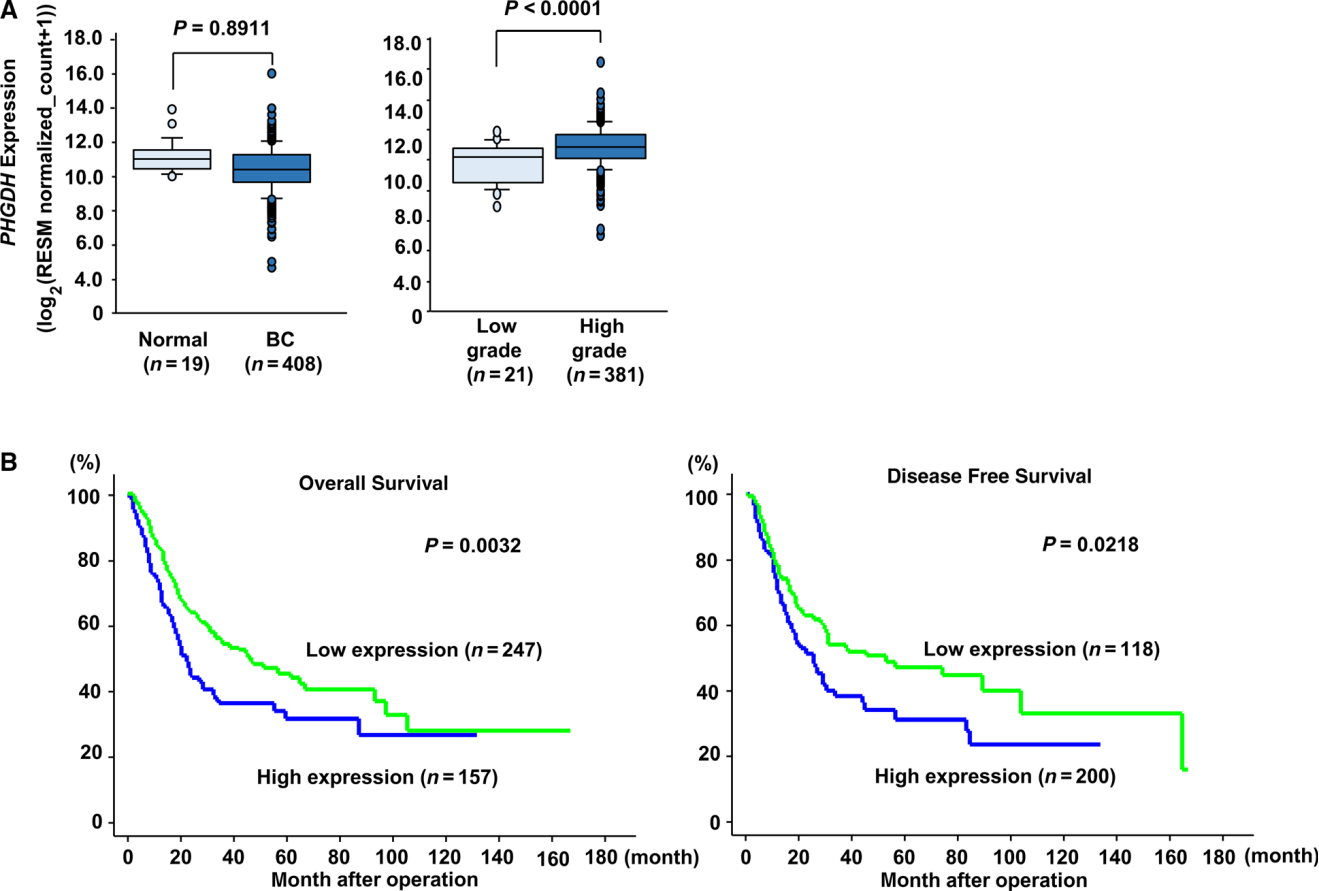
Clinical significance of *PHGDH* expression in BC according to TCGA data. (A) Left: the expression levels of *PHGDH* mRNA in normal human bladders and BCs. Right: the significant positive correlation between *PHGDH* expression and pathological grade (*P* < 0.0001). In BLCA, G1 was for ‘Low grade’ and G3 for ‘High grade’. The Mann–Whitney *U*‐test was performed to assess the statistical relationship, and error bars are represented as mean ± SD. (B) Overall survival (left) and disease‐free survival periods (right) were significantly shortened in patients with high *PHGDH* expression defined as *Z*‐score > 0 compared with those in patients with low *PHGDH* expression defined as *Z*‐score ≤ 0 (*P* = 0.0032 and *P* = 0.0218, respectively). The Kaplan–Meier method and log‐rank test were performed to assess the statistical relationship.

We initially examined the significance of *PHGDH* expression in patients. We used statistical methods to analyze patients in TCGA database. With regard to BC samples and normal samples, there were no significant differences in *PHGDH* mRNA expression levels (*P* = 0.8911; Fig. 1A, left). However, the expression level of *PHGDH* was markedly elevated in high‐grade BC compared with low‐grade disease (*P* < 0.0001; Fig. 1A, right). In addition, patients with high expression of *PHGDH* mRNA defined as *Z*‐score > 0 had poorer overall survival and disease‐free survival in comparison with patients with low expression defined as *Z*‐score ≤ 0 (Log‐rank *P* = 0.0032 and 0.0218, respectively; Fig. 1B). Results were validated in an independent cohort of BC patients (GSE13507) (log‐rank *P* = 0.002307 for overall survival and 0.000262 for disease‐specific survival) (Fig. S1). In addition, the analysis with only high‐grade BC in TCGA cohort (Fig. 1A, right) indicated that patients with high expression of *PHGDH* mRNA also had poorer overall survival and disease‐free survival in comparison with patients with low expression (log‐rank *P* = 0.0081 and 0.0474, respectively; Fig. S2). In TCGA cohort, a Cox univariate analysis showed that *PHGDH* mRNA expression was associated with overall survival and disease‐free survival. In addition, we applied a multivariate Cox proportional hazards model to analyze the data. It showed that the level of *PHGDH* mRNA expression was an independent predictor of both overall and disease‐free survival (Table S3). This finding was validated in a GSE13507 cohort by multivariate Cox analysis that included age, grade, sex, stage, and PHGDH expression; the hazard ratio was 1.866 (1.122–3.104), *P* = 0.0163. We set a cutoff level to 50 years old based on a previous study (Feng *et al*., 2015). Since there have been reports that age and gender are associated with the prognosis for BC patients (Burge and Kockelbergh, 2016; Feng *et al*., 2015; Kucuk *et al*., 2015; Shariat *et al*., 2010), we took the clinical factors into consideration when performing multivariate analysis even if the results were not significant in the univariate analysis of this study cohort.

### 3.2 Effects of PHGDH inhibition in BC cells

**Fig. 2:**
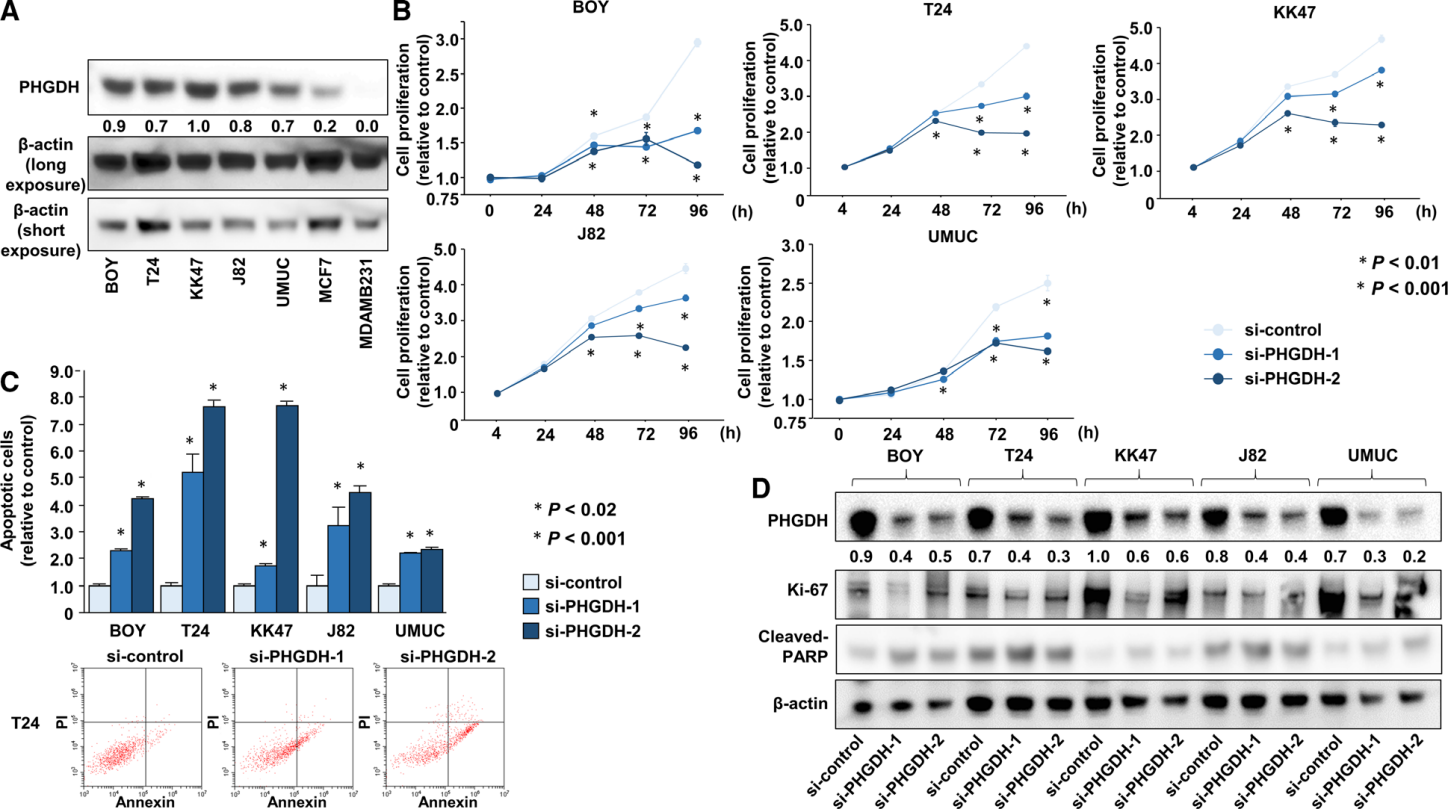
PHGDH inhibition by si‐RNA and inhibitor. (A) Immunoblotting analysis showed that PHGDH expression was elevated in all BC cells compared to breast cancer cells. PHGDH expression values normalized by β‐actin were indicated. (B) Cell proliferation after treatment with *PHGDH* si‐RNA (*P < 0.01, **P < 0.001). The Bonferroni‐adjusted Mann–Whitney *U*‐test was performed to assess the statistical relationship, and error bars are represented as mean ± SD. (C) Apoptosis levels after treatment with *PHGDH* si‐RNA (*P < 0.02, **P < 0.001). The representative quadrant figures of apoptosis assay determined by flow cytometry are shown. Early apoptotic cells can be seen in the bottom right quadrant and late are in the upper right (lower). The Bonferroni‐adjusted Mann–Whitney *U*‐test was performed to assess the statistical relationship, and error bars are represented as mean ± SD. (D) Decreased Ki‐67 and increased cleaved caspase3 levels in si‐*PHGDH*‐transfected BC cells. PHGDH expression values normalized by β‐actin are indicated. Each experiment was carried out in triplicate.

Immunoblotting analyses revealed that PHGDH was markedly increased in BC cells compared with breast cancer cells (MCF7 and MDAMB231), which were used as negative control as shown in the previous study (Possemato *et al*., 2011). MCF7 is an ER+ PR+ HER2− cell line whereas MDAMB231 is a triple negative cell line (Fig. 2A). si‐*PHGDH* transfection of BC cells showed that proliferation was reduced relative to the si‐control (*P < 0.01; **P < 0.001; Fig. 2B).

In addition, there were apoptotic effects in BC cell lines (*P < 0.02; **P < 0.001; Fig. 2C).

**Fig. 3:**
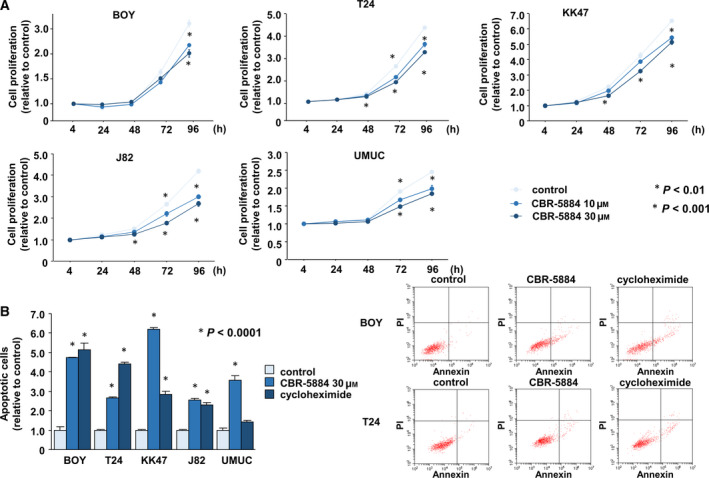
PHGDH inhibition by a PHGDH inhibitor. (A) Cell proliferation after treatment with a PHGDH inhibitor (CBR‐5884) (*P < 0.01, **P < 0.001). The Bonferroni‐adjusted Mann–Whitney *U*‐test was performed to assess the statistical relationship, and error bars are represented as mean ± SD. (B) Apoptosis after treatment with CBR‐5884 (*P < 0.0001). The representative quadrant figures of apoptosis assay determined by flow cytometry are shown. Early apoptotic cells can be seen in the bottom right quadrant and late are in the upper right (right). Each experiment was carried out in triplicate. The Bonferroni‐adjusted Mann–Whitney *U*‐test was performed to assess the statistical relationship, and error bars are represented as mean ± SD.

Using western blots, we found that Ki‐67 was decreased and cleaved PARP levels were increased in BC cells transfected with si‐*PHGDH* compared with the si‐control (Fig. 2D). PHGDH inhibitors (CBR‐5884 and NCT‐503) had similar inhibitory effects on the proliferation of BC cells (*P < 0.01; **P < 0.001; Fig. 3A, Fig. S3). Moreover, CBR‐5884 enhanced the levels of apoptosis in BC cells (*P < 0.0001; Fig. 3B). The positive control utilized 5 μg·mL^−1^ cycloheximide.

### 3.3 PHGDH overexpression in cells under expressing PHGDH

**Fig. 4:**
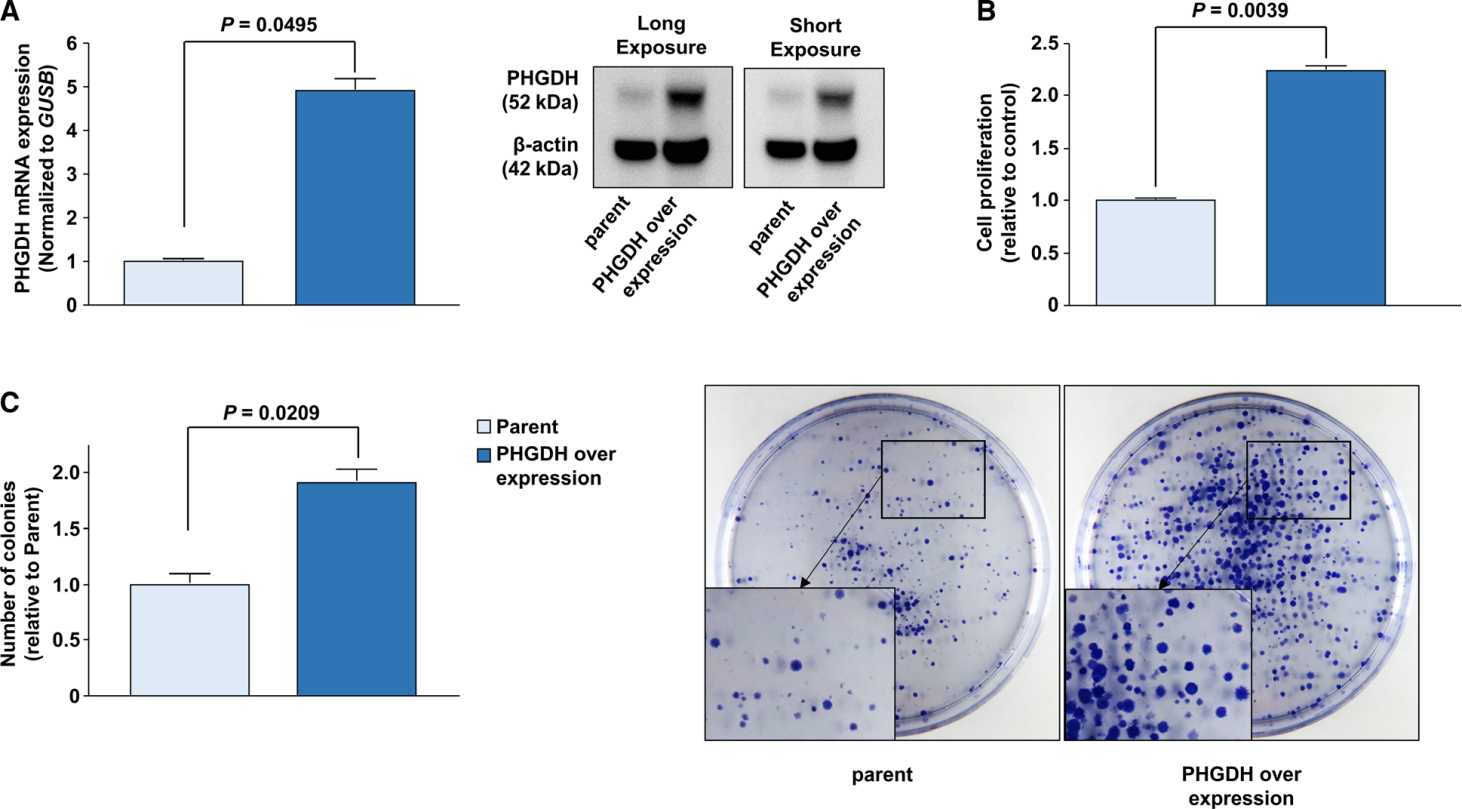
PHGDH overexpression in PHGDH‐downregulated cells. (A) Immunoblotting analysis showed that PHGDH expression was significantly elevated in UMUC cells (*P* = 0.0495). The Mann–Whitney *U*‐test was performed to assess the statistical relationship, and error bars are represented as mean ± SD. (B) Cell proliferation of parental and PHGDH‐overexpressing cells (*P* = 0.0039). The Mann–Whitney *U*‐test was performed to assess the statistical relationship, and error bars are represented as mean ± SD. (C) Representative image of colony formation by parental and PHGDH‐overexpressing UMUC cells (magnification, ×1). The graph showed the ratio of the number of colonies by parental and PHGDH‐overexpressing cell (*P* = 0.0209). Each experiment was carried out in triplicate. The Mann–Whitney *U*‐test was performed to assess the statistical relationship, and error bars are represented as mean ± SD.

Based on the result showing PHGDH expression in BC cells (Fig. 2A), we overexpressed PHGDH in UMUC in which PHGDH was downregulated compared to other BC cell lines (Fig. 4A). The cell proliferative ability (*P* = 0.0039; Fig. 4B) and number of colonies (*P* = 0.0209; Fig. 4C) were significantly increased in UMUC cells in which PHGDH was overexpressed compared to parental cells. For further analyses, we overexpressed PHGDH in MDAMB231 in which PHGDH expression was the lowest (Fig. 2A). The cell proliferative ability (*P* = 0.009; Fig. S4B) and number of colonies (*P* = 0.0209; Fig. S4C) were also significantly increased in PHGDH overexpressed MDAMB231 cells compared to the parental cells.

### 3.4 Inhibition of PHGDH promoted the antitumor effects of gemcitabine and cisplatin

**Fig. 5:**
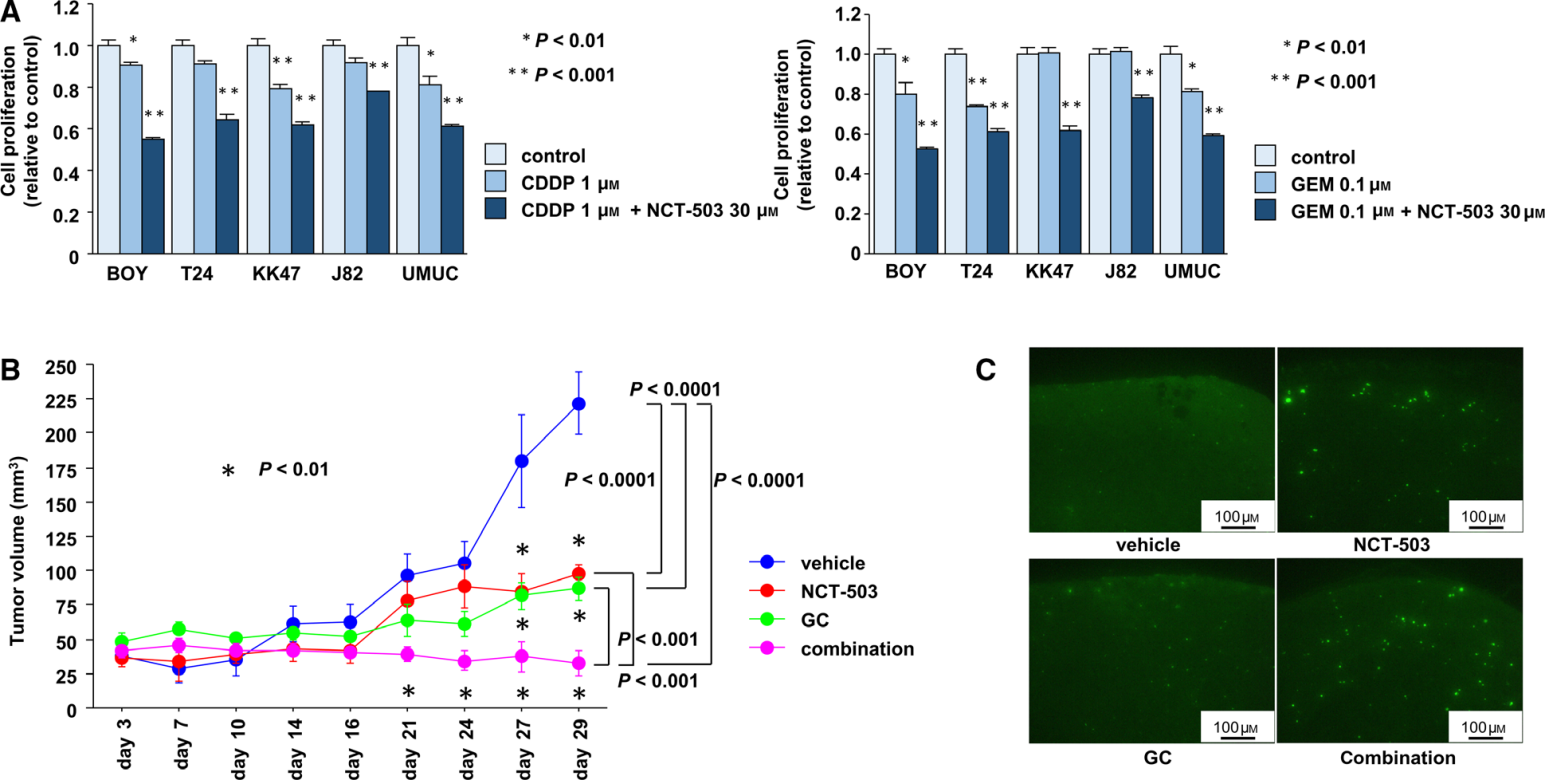
PHGDH inhibition promoted gemcitabine‐ and cisplatin‐induced antitumor effects. (A) Cell proliferation after cisplatin (CDDP) (left) or gemcitabine (GEM) (right) treatment in the absence or presence of a PHGDH inhibitor (NCT‐503) (*P < 0.01, **P < 0.001). The Bonferroni‐adjusted Mann–Whitney *U*‐test was performed to assess the statistical relationship, and error bars are represented as mean ± SD. (B) Time course of tumor volumes formed by subcutaneously injected BOY cells into nude mice. Four groups were examined: (a) vehicle, (b) GC (GEM 150 mg·kg^−1^, i.p., days 7 and 14. CDDP 6 mg·kg^−1^, i.p., days 6 and 13), (c) NCT‐503 (40 mg·kg^−1^, 5 times a week), or (d) a combination of GC and NCT‐503 (*P < 0.01) (*n* = 4 for vehicle or NCT‐503 group, *n* = 6 for GC or combination group). The Bonferroni‐adjusted Mann–Whitney *U*‐test was performed to assess the statistical relationship, and error bars are represented as mean ± SD. (C) Detection of apoptotic cells by TUNEL in tumor xenografts. Bright cells indicate apoptotic cells. Each experiment was carried out in triplicate.

We examined the effects of combining treatments with a PHGDH inhibitor and gemcitabine plus cisplatin. Cell proliferation data showed synergistic effects of tumor suppression compared to that achieved with individual agents (*P < 0.01; **P < 0.001; Fig. 5A, Fig. S5). We next conducted xenograft assays using NCT‐503. We explored this approach because CBR‐5884 was not stable in mouse plasma. In contrast, NCT‐503 has been used with success in xenograft assays (Mullarky *et al*., 2016; Pacold *et al*., 2016). The data showed that tumor growth was markedly reduced in the mice receiving NCT‐503 compared with controls (*P < 0.01; Fig. 5B). Importantly, combined treatments using a PHGDH inhibitor and gemcitabine/cisplatin revealed synergistic inhibition of tumor growth compared to individual agents (Fig. 5B). Relationships among the treatment groups at each time point were analyzed using Bonferroni‐adjusted Mann–Whitney *U*‐tests for the adjustment for multiplicity. TUNEL assays and immunohistochemistry with Ki67 antibodies by using tumor fractions from mice also indicated increased proportions of apoptotic cells and reduced cell proliferation in the tumor from mice treated with NCT‐503 and GC compared to vehicle or single‐agent groups (Fig. 5C, Fig. S6).

### 3.5 Regulatory mechanisms associated with PHGDH in BC

**Fig. 6:**
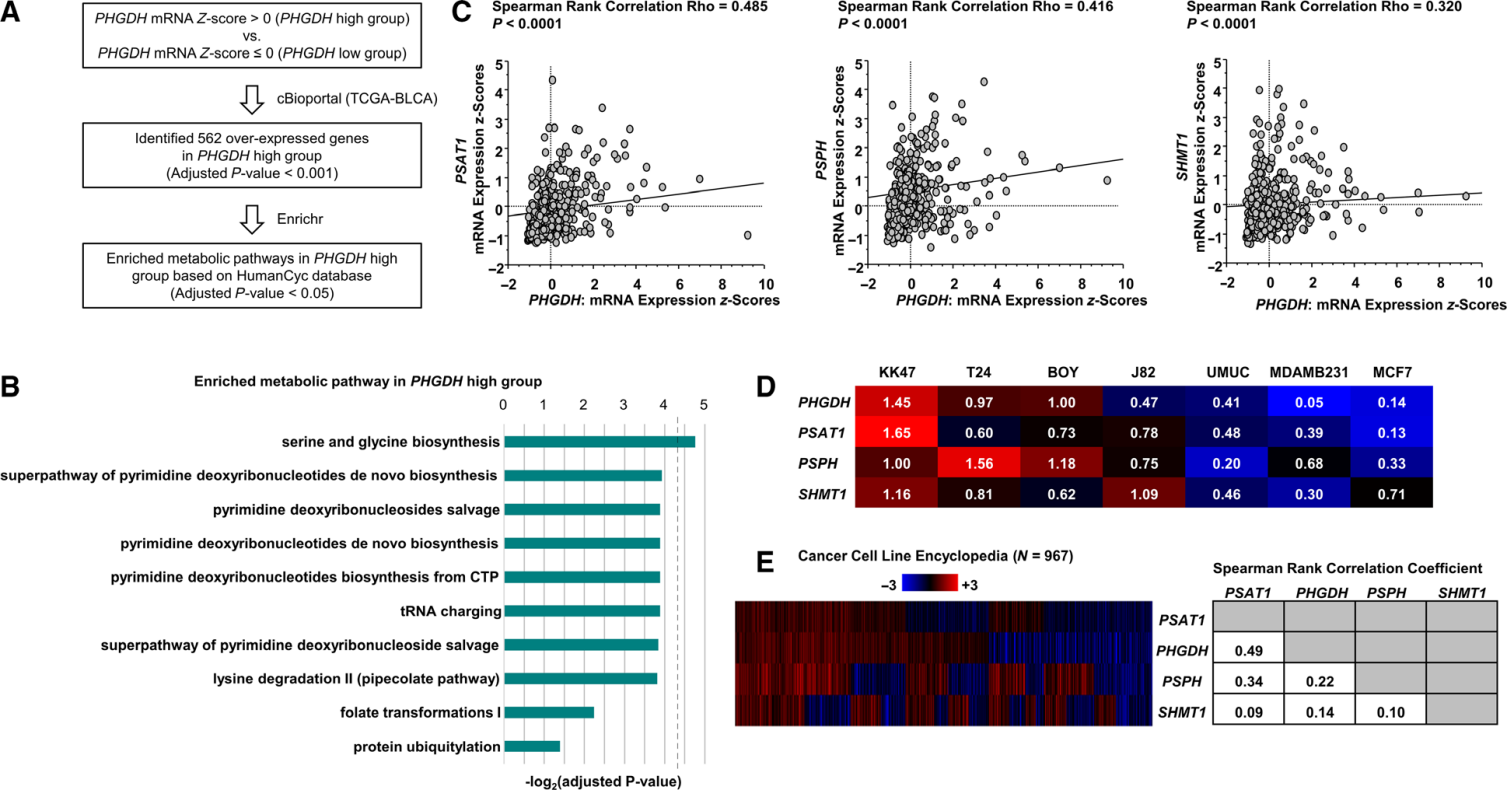
*PHGDH* expression was positively correlated with *PSAT1*, *PSPH*, and *SHMT1* expression in cancer cells. (A) Flowchart of the strategy for identification of enriched metabolic pathways in the *PHGDH* high group. (B) Pathway analysis by Enrichr was performed using the HumanCyc Database. Fisher's exact test, followed by the Benjamini–Hochberg procedure was performed to assess the statistical relationship. (C) Spearman's rank test demonstrated that *PHGDH* and *PSAT1*, *PSPH* or *SHMT1* mRNA expression levels were positively correlated (each *P* < 0.0001). (D) Expression of *PSAT1*, *PSPH*, or *SHMT1* mRNA was positively correlated with *PHGDH* mRNAs expression in BC and breast cancer cell lines. Each value indicates the relative ratio compared to *PHGDH* mRNA expression in BOY cells. (E) Positive correlations between the expression of *PHGDH* and *PSAT1*, *PSPH* or *SHMT1* mRNAs were observed in other cancer cell lines (left). Each value indicates the correlation coefficient of the heat map (right). Spearman's rank test was performed to assess the statistical relationship.

**Fig. 7:**
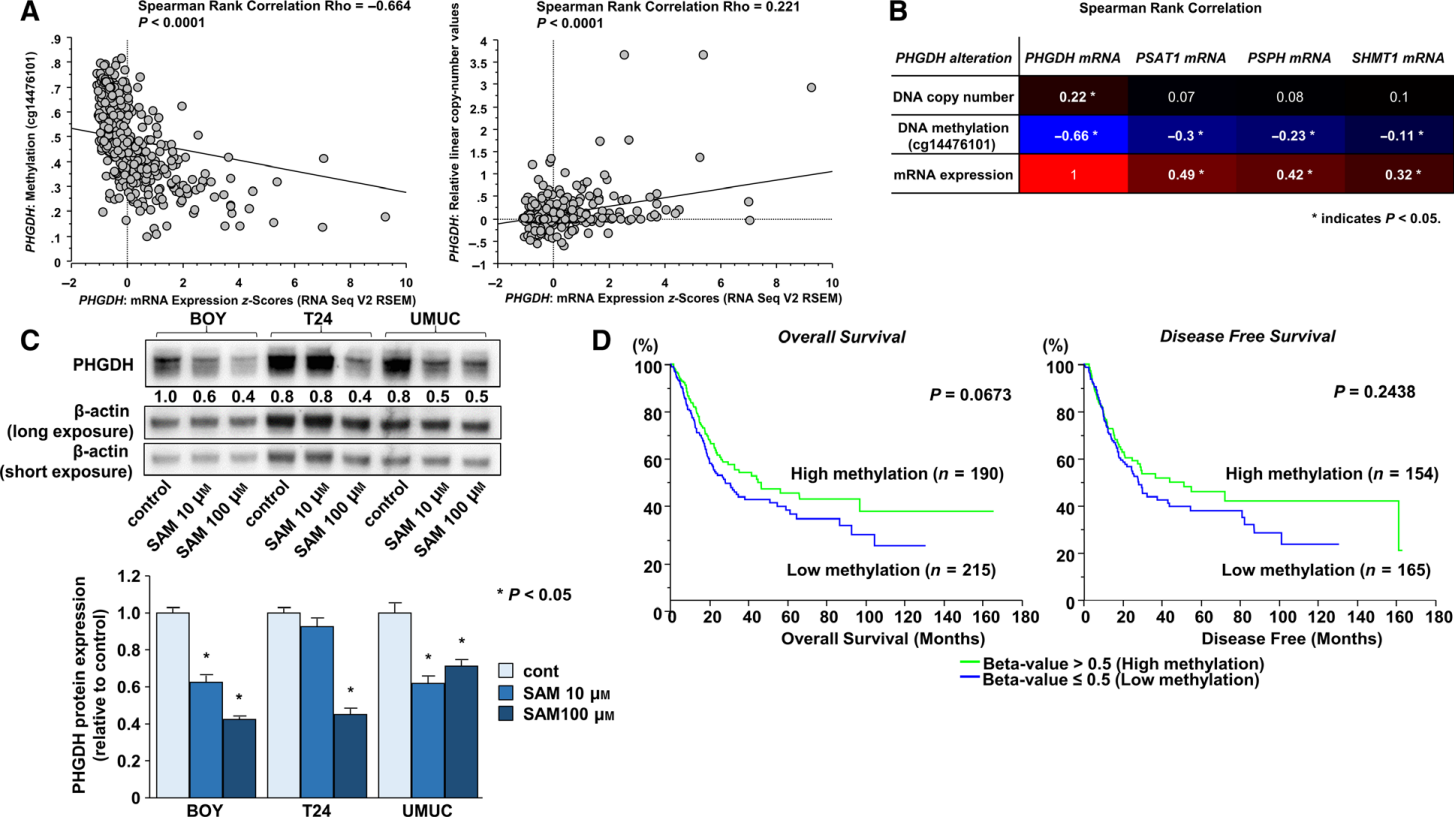
Relationship between *PHGDH* mRNA expression and DNA methylation or copy number. (A) Spearman's rank test indicated a negative correlation between *PHGDH* mRNA expression and DNA methylation (left), and a positive correlation between *PHGDH* mRNA expression and its copy number (right). (B) Correlation between *PHGDH*, *PSAT1*, *PSPH* or *SHMT1* mRNA expression and *PHGDH* copy number, methylation, or mRNA expression. Spearman's rank test was performed to assess the statistical relationship. (C) *PHGDH* expression was decreased by SAM in BC cells. PHGDH expression values are normalized by β‐actin as indicated. Experiments were carried out in triplicate. The Bonferroni‐adjusted Mann–Whitney *U*‐test was performed to assess the statistical relationship, and error bars are represented as mean ± SD. (D) Overall survival (left) and disease‐free survival periods (right) were shortened in patients with low methylation of *PHGDH* compared with those in patients with high methylation (0.0673 and *P* = 0.2438, respectively). The Kaplan–Meier method and log‐rank test were performed to assess the statistical relationship.

Previous study showed that hypoxia coordinately induced PHGDH, PSAT1, and PSPH expression, which are located downstream from *PHGDH* (Samanta *et al*., 2016). Then, we used cBioPortal to analyze 562 differentially overexpressed genes in the *PHGDH* high group (mRNA *Z*‐score > 0) compared with the *PHGDH* low group (mRNA *Z*‐score ≤ 0) in TCGA BLCA cohort (Fig. 6A) to check whether genes on the serine and glycine biosynthesis pathway were accelerated in concert with *PHGDH* expression. The serine and glycine biosynthesis pathway was found prominently at the top of the list by Enrichr pathway analysis (Fig. 6B). Using TCGA BLCA database, we observed significant positive correlations between *PHGDH* and each of the genes (*P* < 0.0001; Fig. 6C). These correlations were supported by the results from cancer cell lines. Those data showed that expression of *PSAT1*, *PSPH*, or *SHMT1* mRNAs was positively correlated with *PHGDH* mRNA expression (Fig. 6D,E). We also checked the regulatory mechanisms associated with PHGDH expression. According to TCGA BLCA database, the *PHGDH* DNA methylation site harboring cg14476101 and mRNA expression showed significant negative correlations (ρ = −0.664; *P* < 0.0001; Fig. 7A left, and B), and *PHGDH* DNA copy number and mRNA expression showed significantly positive correlations (ρ = 0.221; *P* < 0.0001; Fig. 7A right, B). In addition, *PHGDH* expression was decreased when BC cell lines were treated for 48 h with SAM, a biological methyl donor (*P* < 0.05; Fig. 7C). Finally, overall (left) and disease‐free survivals (right) were somewhat shorter in patients (Fig. 1B) with high *PHGDH* and reduced methylation relative to patients with high methylation of *PHGDH* (*P* = 0.0673 and *P* = 0.2438, respectively; Fig. 7D).

## 4 Discussion

There have been reports that PHGDH inhibition or depletion leads to apoptosis in various cancers (Jing *et al*., 2015; Ou *et al*., 2015; Samanta *et al*., 2016). To the best of our knowledge, this analysis is the first to demonstrate its role in BC and its potential as a prognostic marker. In addition, we demonstrated that combination treatment using GC therapy and PHGDH inhibition had an additive apoptotic effect *in vivo* without weight loss and obvious side effects. Combination therapy with gemcitabine and/or cisplatin for BC has been reported by several researchers (Zeng *et al*., 2017). They showed that pictilisib, an inhibitor of PI3K, improved the antitumor effects of cisplatin and gemcitabine in human BC, both *in vitro* and *in vivo*. This was achieved by reducing the phosphorylation of ribosomal protein S6. Another combination was reported by Grivas *et al*. (2013). They found that dacomitinib, a tyrosine kinase inhibitor of EGFR, HER2 and HER4, could be combined with gemcitabine–cisplatin chemotherapy to improve the response achieved by gemcitabine–cisplatin alone. In terms of apoptosis enhancement, which was observed in this study, Smac mimetics (second mitochondria‐derived activator of caspases) and 1,25 dihydroxyvitamin D3 (1,25 D3), the most active vitamin D metabolite, showed synergistic effects against BCs in combination with GC treatment (Lee *et al*., 2013; Ma *et al*., 2010). The main mechanism of GC therapy is the induction of tumor cell apoptosis, and the inactivation of apoptotic pathways is a crucial and common mechanism for tumor cell resistance to GC (Bergman *et al*., 2002; Wang and Lippard, 2005). Thus, our finding that PHGDH silencing plus GC treatment had additive antitumor effects through induction of apoptosis was reasonable. Inhibition of PHGDH might be a candidate therapeutic option to be combined with GC treatment. GC therapy is well known to cause grades 3 and 4 toxicities as defined by the World Health Organization (von der Maase *et al*., 2000). We anticipate that the combination of GC therapy and PHGDH inhibition might reduce the current dose of GC therapy, thereby avoiding severe adverse events and toxic death. For that reason, we suggest that these inhibitors or next‐generation PHGDH inhibitors should be used in clinical trials in the near future.

Several reports have shown that DNA amplification of *PHGDH* underlies the high expression of PHGDH in various types of cancers (melanoma, breast cancer, and clear cell renal cell carcinoma) (Locasale *et al*., 2011; Possemato *et al*., 2011). However, this is the first report showing that PHGDH expression was regulated by methylation. Truong *et al*. studied blood samples of patients with venous thromboembolism. Their analysis showed that cg14476101, which is located in the first intron of *PHGDH*, is a methylation site. In monocytes, elevated methylation at that site was significantly associated with reduced *PHGDH* expression according to database eMS (Liu *et al*., 2013; Truong *et al*., 2017). In addition, Aslibekyan *et al*. (2015) reported that cg14476101 was located within a methylated region in noncancerous blood samples. Therefore, our finding that *PHGDH* hypomethylation was associated with poor prognosis based on characterization of cg14476101 is plausible. Moreover, we showed that introduction of a biological methyl donor into BC cells decreased PHGDH expression levels. Serine biosynthesis is the main pathway for the production of SAM, a substrate used in the methylation of lipids, proteins, RNA, DNA, and metabolites (Maddocks et al., 2016; Mentch *et al*., 2015). Moreover, PHGDH inhibition did not influence reactive oxygen species levels in BC (data not shown). Thus, we speculate that the acceleration of serine biosynthesis through increased PHGDH expression in BC might upregulate production of cell constituents (proteins, nucleic acids and lipids) that are necessary for cancer cell growth (Amelio *et al*., 2014). Moreover, we speculate that targeting PHGDH could suppress serine biosynthesis, leading to the inhibition of cancer cell viability through downregulation of vital cell constituents. Additional studies will be required to enhance our understanding of the underlying pathways of cancer metabolism.

## 5 Conclusions

Our investigation of BC revealed that the expression level of *PHGDH* was correlated with tumor grade and prognosis. We believe that this is the first report to show that *PHGDH* may be a prognostic marker predicting BC patient survival. In addition, PHGDH inhibitors significantly inhibited cancer cell growth, and synergistic effects with GC treatment were observed both *in vitro* and *in vivo* in BC. Furthermore, this is the first report indicating that PHGDH expression might be accelerated by hypomethylation in cancers. Taken together, our data may provide insights into the mechanisms underlying BC and suggest new therapeutic approaches and biomarkers.

## Conflict of interest

The authors declare no conflict of interest. NN is an employee of MSD. KK is a subsidiary of Merck & Co., Inc. and reports personal fees from MSD K. K. outside this study.

## Author contributions

HY conceived and designed the study. None of the authors involved in development of methodology. HY, YO, NN, MY, SS, KK, and MT acquired the data. HY, HE, and NN analyzed and involved in interpretation of data. HY, HE, and NN wrote, reviewed and/or revised the manuscript. None of the authors involved in administrative, technical, or material support. HE, ST, and MN supervised the study.

## Supporting information


**Fig. S1.** Clinical significance of *PHGDH* expression in BC with GSE13507 cohort. Overall survival (left) and disease‐free survival periods (right) were significantly shortened in patients with high PHGDH expression compared with those in patients with low PHGDH expression (*P* = 0.002307 and *P* = 0.000262, respectively). The Kaplan–Meier method and log‐rank test were performed to assess the statistical relationship.

**Fig. S2.** Clinical significance of *PHGDH* expression in BC with high grade in TCGA data. Overall survival (left) and disease‐free survival periods (right) were significantly shortened in patients with high PHGDH expression compared with those in patients with low PHGDH expression (*P* = 0.0081 and *P* = 0.0474, respectively). The Kaplan–Meier method and log‐rank test were performed to assess the statistical relationship.

**Fig. S3.** PHGDH inhibition by PHGDH inhibitor (NCT‐503). Cell proliferation assay after treatment with a PHGDH inhibitor (NCT‐503). (*, *P* < 0.01; * *, *P* < 0.001). Bonferroni‐adjusted Mann–Whitney U‐test was performed to assess the statistical relationship, and error bars are represented as mean ± SD (*n* = 3).

**Fig. S4.** PHGDH overexpression in PHGDH‐downregulated cells. (A) Immunoblotting analysis showed that PHGDH expression was dramatically elevated in MDAMB231 cells. Error bars are represented as mean ± SD (*n* = 3). (B) Cell proliferation of parental and PHGDH‐overexpressing cells (*P* = 0.009). Error bars are represented as mean ± SD (*n* = 5). (C) Representative image of colony formation by parental and PHGDH‐overexpressing MDAMB231 cells (magnification, x 1). The graph showed the ratio of the number of colonies by parental and PHGDH‐overexpressing cells (*P* = 0.0209). Error bars are represented as mean ± SD (*n* = 4). The Mann–Whitney U‐test was performed to assess the statistical relationship on each experiment.

**Fig. S5.** PHGDH inhibition promoted a gemcitabine‐ and cisplatin‐induced antitumor effect. Cell proliferation after treatment with cisplatin (left) or gemcitabine (right) in the absence or presence of a PHGDH inhibitor (CBR‐503). Bonferroni‐adjusted Mann–Whitney U‐test was performed to assess the statistical relationship, and error bars are represented as mean ± SD (*n* = 3).

**Fig. S6.** Ki67‐positive cells were decreased by PHGDH inhibition and gemcitabine/cisplatin compared to vehicle or single‐agent groups. Ki67‐positive cells were calculated from independent tumor sections per group and expressed as the mean ± SD (*, *P* < 0.05) (*n* = 4 for vehicle or GC group, *n* = 3 for NCT‐503 or combination group). (magnification, x 400). Bonferroni‐adjusted Mann–Whitney U‐test was performed to assess the statistical relationship.

**Table S1.** Clinical and demographic characteristics of TCGA Bladder urothelial carcinoma (BLCA) samples categorized based on PHGDH expression level.
**Table S2.** Clinical and demographic characteristics of GSE13507 bladder cancer cohort categorized based on PHGDH expression level.
**Table S3.** Univariate and multivariate analysis in BLCA cohort database.
