# Supplementary figures and images for: Characterization of PHGDH expression in bladder cancer: potential targeting therapy with gemcitabine/cisplatin and the contribution of promoter DNA hypomethylation
Source: Mol Oncol. 2020 Jun 20;14(9):2190–202. doi: 10.1002/1878-0261.12697 (PMC7463350; doi:10.1002/1878-0261.12697)

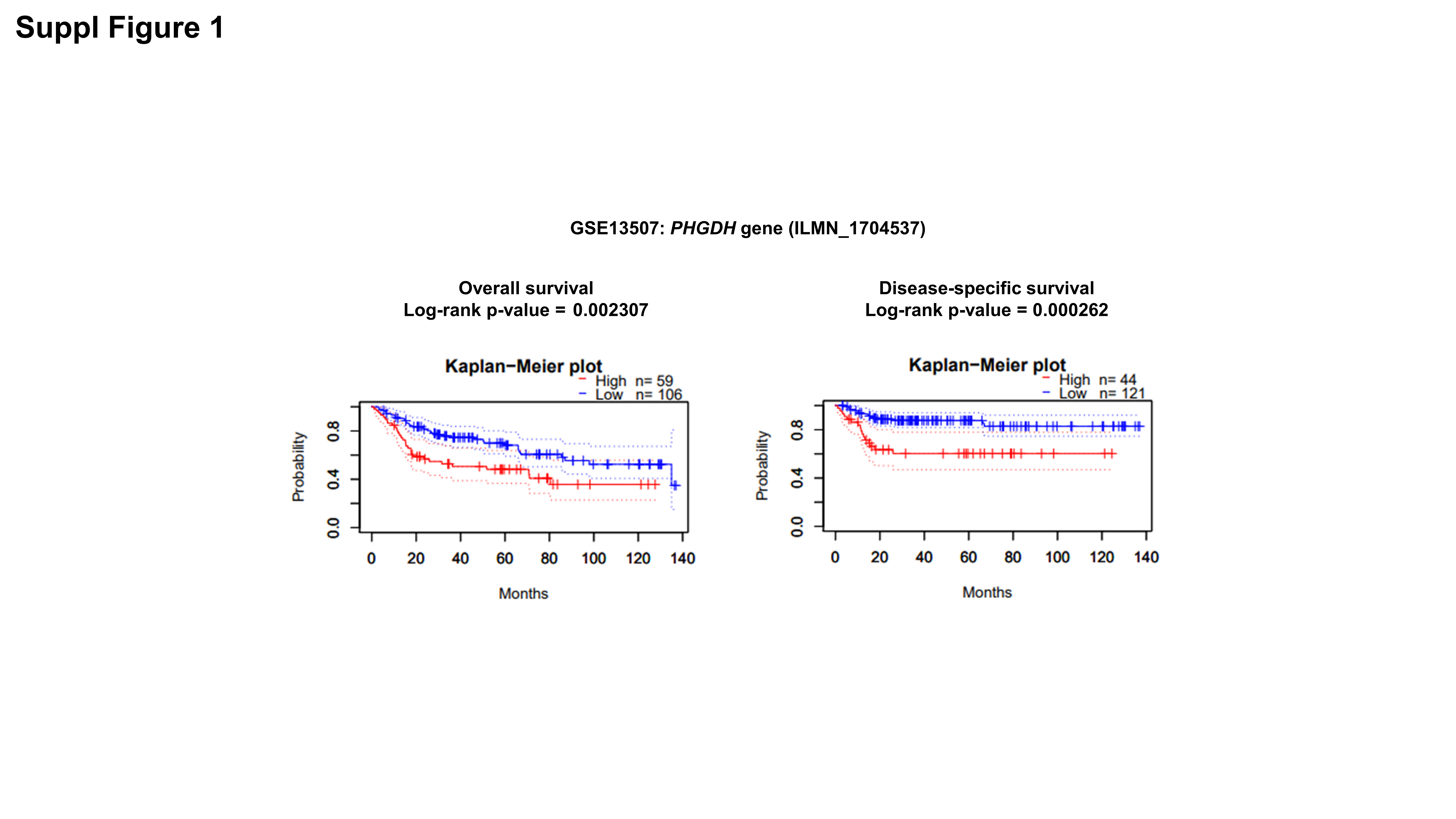

Supplement: Supplementary file 1 — Fig. S1. Clinical significance of PHGDH expression in BC with GSE13507 cohort. Overall survival (left) and disease‐free survival periods (right) were significantly shortened in patients with high PHGDH expression compared with those in patients with low PHGDH expression (P = 0.002307 and P = 0.000262, respectively). The Kaplan–Meier method and log‐rank test were performed to assess the statistical relationship. [file MOL2-14-2190-s001.tif]

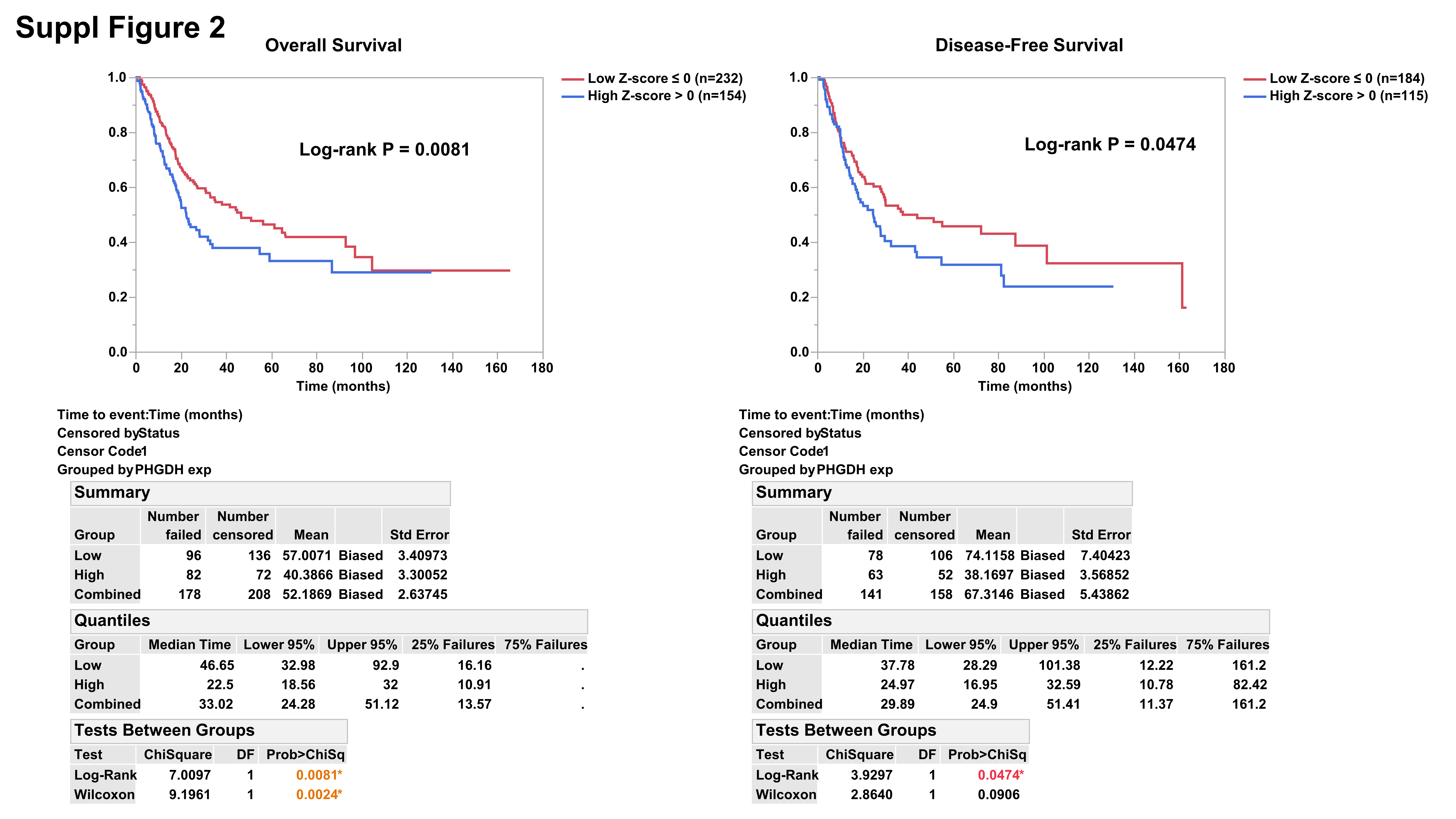

Supplement: Supplementary file 2 — Fig. S2. Clinical significance of PHGDH expression in BC with high grade in TCGA data. Overall survival (left) and disease‐free survival periods (right) were significantly shortened in patients with high PHGDH expression compared with those in patients with low PHGDH expression (P = 0.0081 and P = 0.0474, respectively). The Kaplan–Meier method and log‐rank test were performed to assess the statistical relationship. [file MOL2-14-2190-s002.tif]

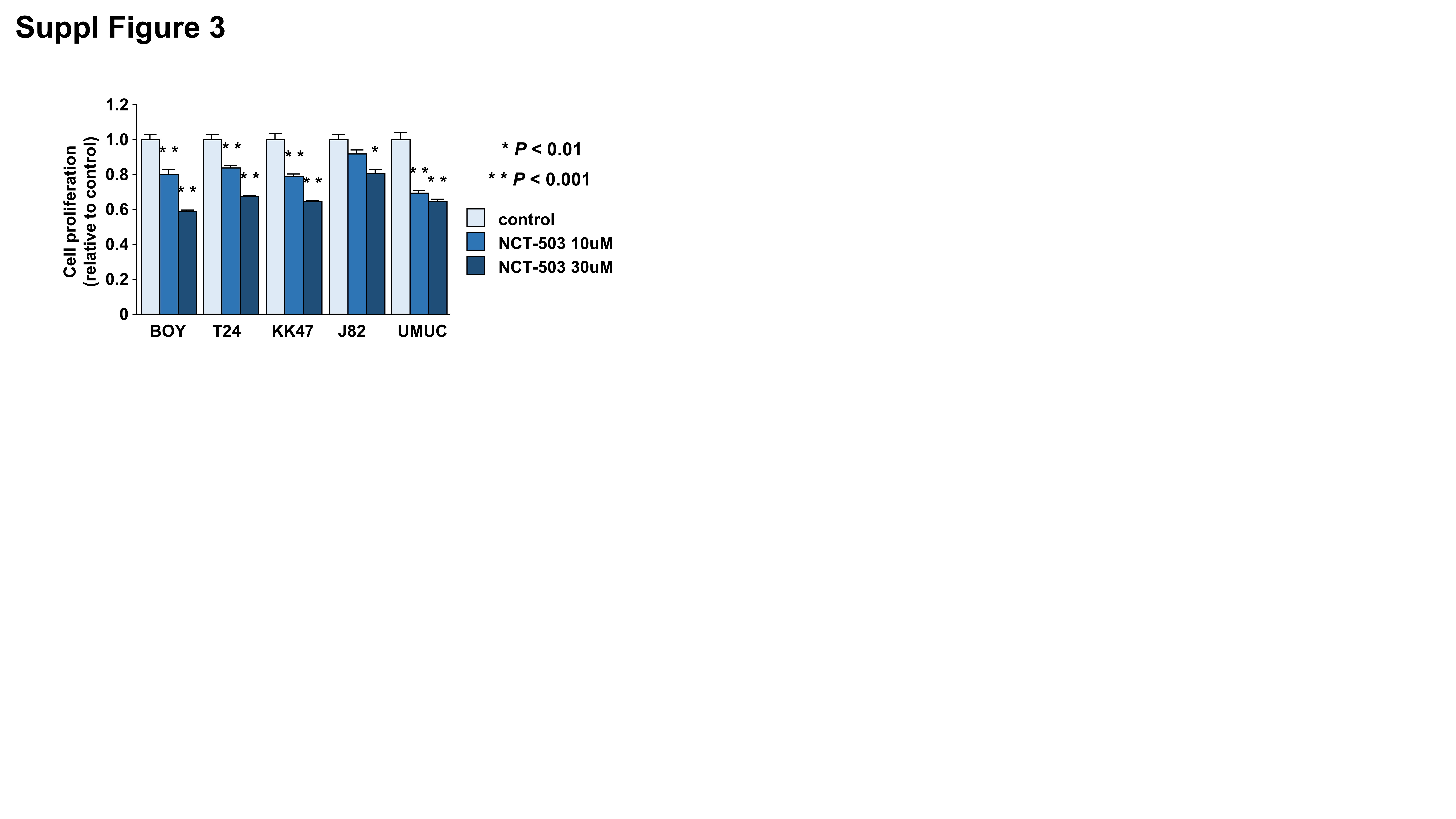

Supplement: Supplementary file 3 — Fig. S3. PHGDH inhibition by PHGDH inhibitor (NCT‐503). Cell proliferation assay after treatment with a PHGDH inhibitor (NCT‐503). (*, P < 0.01; * *, P < 0.001). Bonferroni‐adjusted Mann–Whitney U‐test was performed to assess the statistical relationship, and error bars are represented as mean ± SD (n = 3). [file MOL2-14-2190-s003.tif]

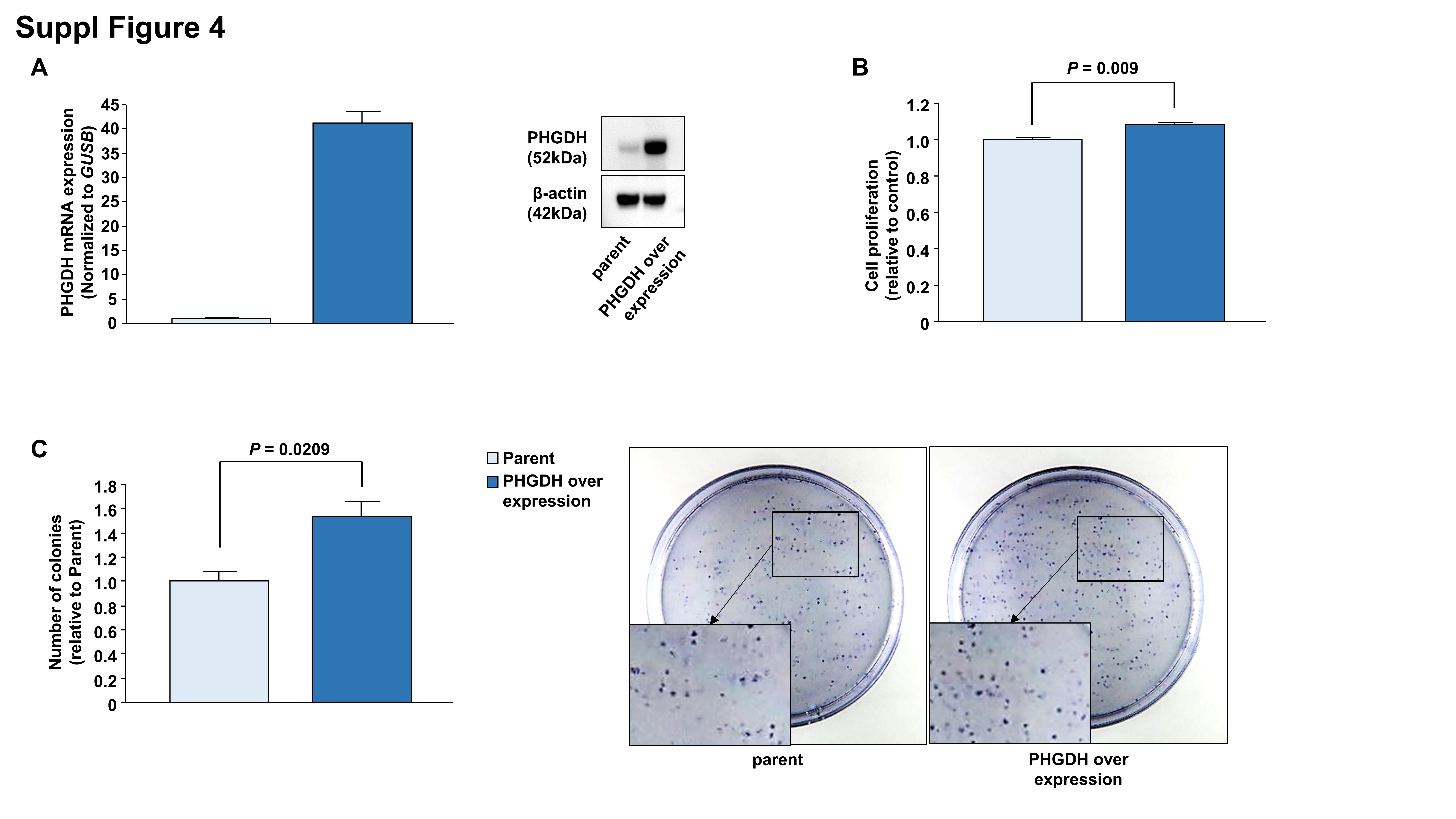

Supplement: Supplementary file 4 — Fig. S4. PHGDH overexpression in PHGDH‐downregulated cells. (A) Immunoblotting analysis showed that PHGDH expression was dramatically elevated in MDAMB231 cells. Error bars are represented as mean ± SD (n = 3). (B) Cell proliferation of parental and PHGDH‐overexpressing cells (P = 0.009). Error bars are represented as mean ± SD (n = 5). (C) Representative image of colony formation by parental and PHGDH‐overexpressing MDAMB231 cells (magnification, x 1). The graph showed the ratio of the number of colonies by parental and PHGDH‐overexpressing cells (P = 0.0209). Error bars are represented as mean ± SD (n = 4). The Mann–Whitney U‐test was performed to assess the statistical relationship on each experiment. [file MOL2-14-2190-s004.tif]

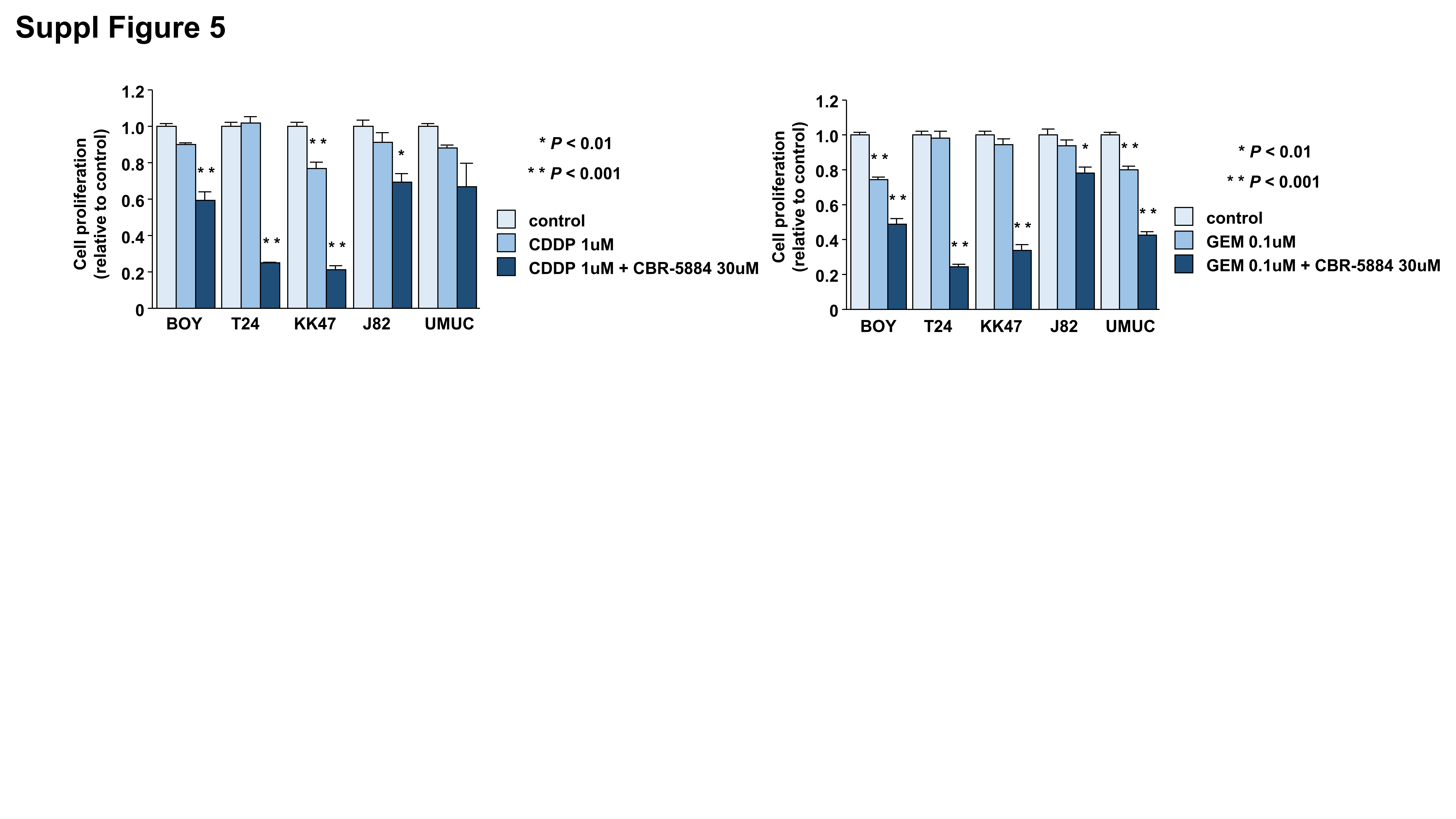

Supplement: Supplementary file 5 — Fig. S5. PHGDH inhibition promoted a gemcitabine‐ and cisplatin‐induced antitumor effect. Cell proliferation after treatment with cisplatin (left) or gemcitabine (right) in the absence or presence of a PHGDH inhibitor (CBR‐503). Bonferroni‐adjusted Mann–Whitney U‐test was performed to assess the statistical relationship, and error bars are represented as mean ± SD (n = 3). [file MOL2-14-2190-s005.tif]

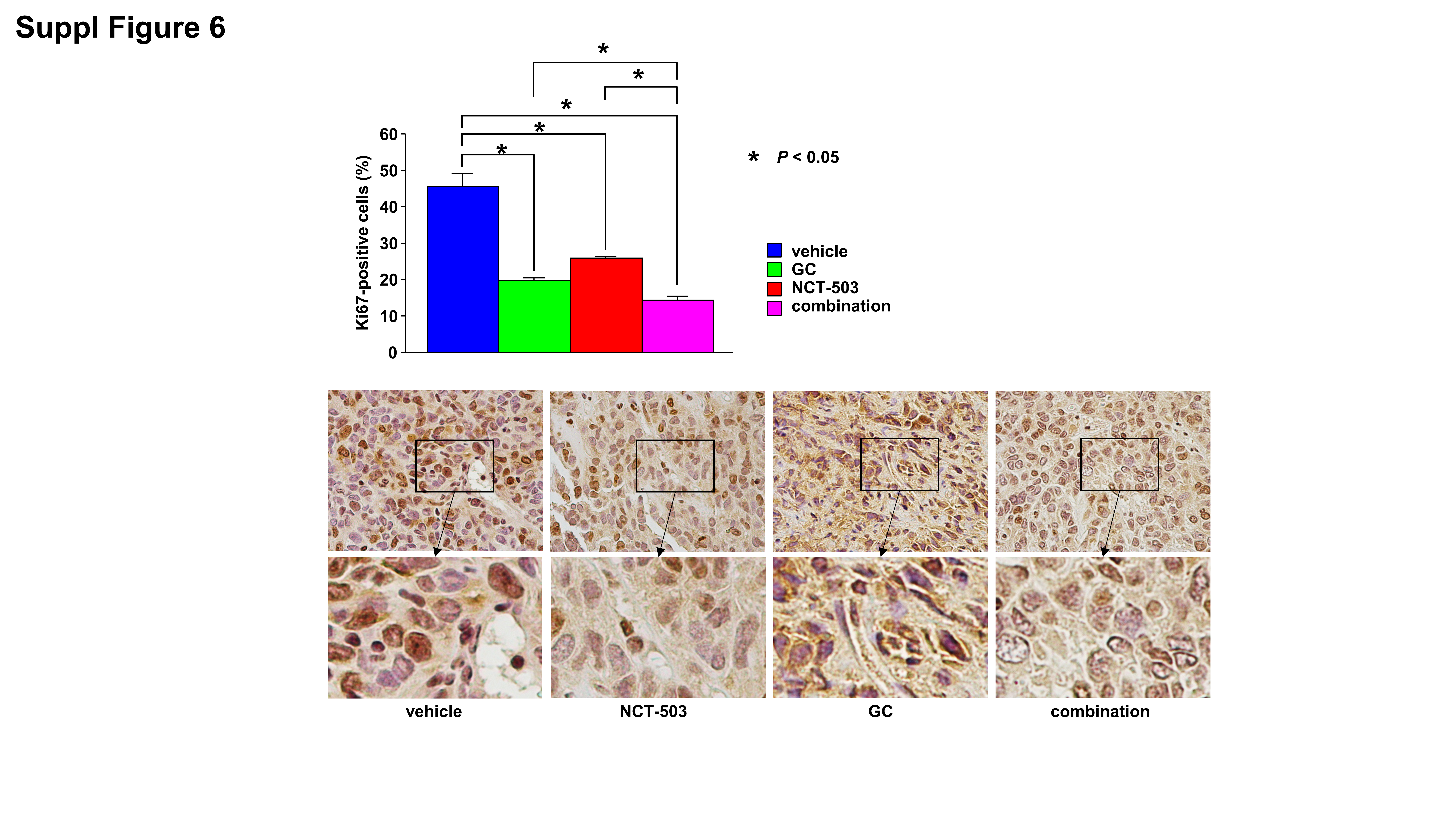

Supplement: Supplementary file 6 — Fig. S6. Ki67‐positive cells were decreased by PHGDH inhibition and gemcitabine/cisplatin compared to vehicle or single‐agent groups. Ki67‐positive cells were calculated from independent tumor sections per group and expressed as the mean ± SD (*, P < 0.05) (n = 4 for vehicle or GC group, n = 3 for NCT‐503 or combination group). (magnification, x 400). Bonferroni‐adjusted Mann–Whitney U‐test was performed to assess the statistical relationship. [file MOL2-14-2190-s006.tif]
