# Supplementary material for: Characterization of PHGDH expression in bladder cancer: potential targeting therapy with gemcitabine/cisplatin and the contribution of promoter DNA hypomethylation
Source: Mol Oncol. 2020 Jun 20;14(9):2190–202. doi: 10.1002/1878-0261.12697 (PMC7463350; doi:10.1002/1878-0261.12697)
Supplement: Supplementary file 7 — Table S1. Clinical and demographic characteristics of TCGA Bladder urothelial carcinoma (BLCA) samples categorized based on PHGDH expression level. Table S2. Clinical and demographic characteristics of GSE13507 bladder cancer cohort categorized based on PHGDH expression level. Table S3. Univariate and multivariate analysis in BLCA cohort database. [file MOL2-14-2190-s007.docx]

**Supplemental Table 1: Clinical and demographic characteristics of TCGA Bladder urothelial carcinoma (BLCA) samples categorized based on PHGDH expression level**

**Supplemental Table 2: Clinical and demographic characteristics of GSE13507 bladder cancer cohort categorized based on PHGDH expression level**

**Supplemental Table 3: Univariate and multivariate analysis in BLCA cohort database**

**Supplemental Table 1: Clinical and demographic characteristics of TCGA Bladder urothelial carcinoma (BLCA) samples categorized based on PHGDH expression level**

**Supplemental Table 2: Clinical and demographic characteristics of GSE13507 bladder cancer cohort categorized based on PHGDH expression level**

**Supplemental Table 3: Univariate and multivariate analysis in BLCA cohort database**

**Overall survival**

**Disease-free survival**
